# Conductive bacterial cellulose by *in situ* laccase polymerization of aniline

**DOI:** 10.1371/journal.pone.0214546

**Published:** 2019-04-15

**Authors:** Euijin Shim, Jing Su, Jennifer Noro, Marta A. Teixeira, Artur Cavaco-Paulo, Carla Silva, Hye Rim Kim

**Affiliations:** 1 Department of Clothing and Textiles, Sookmyung Women’s University, Yongsan-gu, Seoul, South Korea; 2 International Joint Research Laboratory for Textile and Fiber Bioprocesses, Jiangnan University, Wuxi, China; 3 Centre of Biological Engineering, University of Minho, Campus of Gualtar, Braga, Portugal; 4 Department of Textile Engineering, Campus of Azurém, Guimarães, Portugal; 5 Research Institute of ICT Convergence, Sookmyung Women’s University, Seoul, Korea; Institute of Materials Science, GERMANY

## Abstract

Conductive and colored bacterial cellulose (BC) was developed by entrapment of polyaniline (PANi) onto dry BC membranes. The polyaniline was produced by *in situ* green polymerization of aniline by *Myceliophthora thermophila* laccase at pH = 4, 25°C, in the presence of a mediator, 1-hydroxybenzotriazol (HBT), using two different reactors, a water bath (WB) and an ultrasonic bath (US). MALDI-TOF and ^1^H NMR characterization of the experiment solutions confirmed the efficient polymerization of aniline by laccase. The dried BC membranes with entrapped polyaniline showed electrical conductive behavior and strong coloration, opening novel routes for the exploitation of functionalized bacterial cellulose as a green material for technical textiles, wearables, and other applications.

## Introduction

Bacterial cellulose (BC) is a natural polymer with intrinsic 3D network structure, high porosity and ultrafine mechanical properties [1–4]. The *in situ* incorporation of different compounds into the BC fibrils is often used to create novel types of nanocomposites, combining the intrinsic BC structural properties with the additional properties given by the components incorporated [5–9]. From the variety of compounds that might be incorporated into BC, the conductive substrates represent an important market niche however still poorly explored. Conductive polymers when applied in electrochemical systems, would confer to BC products excellent redox properties, high environmental stability and trouble-less synthesis [10, 11].

Polyaniline, typically prepared by the chemical oxidation of aniline, has been widely used as a conducting polymer due to its easy synthesis, low monomer cost, excellent conductivity, high chemical resistance and thermal stability, compared to other conducting polymers [12,13]. The conjugation mechanism of polyaniline is unique among other conducting polymers owing to a combination of benzenoid and quinoid rings leading to three different oxidation states. Both chemical and electrochemical polymerization methods used for aniline polymerization involve harsh conditions like oxidant ammonium peroxydisulfate, potassium dichromate or ferric chloride, in low acidic solutions (pH 0–1), for the formation of head-to-tail structure of polyaniline [12, 14]. When laccase (benzenediol:oxygen oxidoreductase, EC 1.10.3.2) is used as reaction catalyst, it allows the oxidative polymerization of aniline under environmentally friendly and relatively mild conditions, i.e., slightly acidic aqueous solution (pH 4–6) and room temperature, without the formation of toxic byproducts such as benzidine [9]. Compared with other enzymes like peroxidases, laccase catalysis of aniline is not only environmentally friendly but also lacks a stepwise addition of diluted hydrogen peroxide to the reaction medium [12, 15,16]. Redox mediators can enhance the enzymatic polymerization by mediating the oxidation reaction between the substrate and the enzyme [14,17,18]. Efficient mediators that share the structural feature of being N-OH derivatives, such as 1-hydroxybenzotriazole (HBT), have been studied. The oxidation of this type of mediators by laccase generates a highly reactive *N*-oxyl radical, owing to the enzymatic removal of an electron followed by release of a proton [19, 20].

The polymers like polyaniline, synthesized by laccase, have been described as potential dyestuff for materials green coloration. The products of enzymatic polymerization of some substrates undergo coloration and are able to confer color to the substrates when applied *in situ* [21, 22], as can be found in literature [16, 23, 24]. Previously Song *et al*. explored the biocoloration of BC by laccase-assisted polymerization of phenolics and found that this substrate is easily colored by the new polymers formed revealing leaching resistance when washed [24]. Another study was conducted by Jing et al. describing the production of colored and conductive cotton by laccase-assisted polymerization aniline [25]. Despite all the efforts, reactional improvements, including the use of mediators, temperature variations, different reactors, are required to enhance the coating yield and consequently the performance of BC materials for uses as colorful conducting materials [26]. Our aim in this study was to produce a BC conductive material through the *in situ* polymerization of aniline by laccase. The polymerization of aniline was performed in an acidic medium in the presence of a mediator, 1-hydroxybenzotriazol (HBT), without addition of initiator. The role of ultrasound on the enzymatic-assisted polymerization of aniline was also evaluated. The polymerization was followed during time by UV/Visible spectroscopy and the new oligomers/polymers produced were characterized by ^1^H NMR and MALDI-TOF spectroscopy. The conductivity was evaluated using two-probe method and the spectral values of colored BC samples were spectrophotometrically evaluated.

## Experimental

### Materials

Commercially available bacterial cellulose (BC) gel (tea fungus, GetKombucha.com, Culver, CA, USA), was used to produce BC samples. Glucose (Duksan Pure Chemicals Co., Seoul, Korea) was used as carbon source. A mixture of yeast extract (Becton, Dickinson and Company, Sparks, USA) and peptone (Becton, Dickinson and company, Sparks, USA) were used as nitrogen sources. The following chemicals were used without further purification: acetic acid (Duksan Pure Chemicals Co., Seoul, Korea), sodium acetate (Sigma, Saint Louis, USA), hydrogen peroxide (Duksan Pure Chemicals., Korea), aniline (Junsei Chemicals Co., Tokyo, Japan), 1-hydroxybenzotriazole–HBT (Sigma, Saint Louis, USA). Laccase (EC 1.10.3.2.) from *Myceliophthora thermophila* was applied at pH 4 (0.1 M acetate buffer). Citric acid monohydrate (Sigma, Saint Louis, USA), sodium phosphate dibasic dehydrate (Riedel-de Haën, Seelze, Germany).

### Preparation of bacterial cellulose (BC) gel samples

BC gel samples were produced and pretreated according to the previously reported methodology [27]. The Hestrin-Schramm (HS) medium was prepared adding glucose as carbon source (20 g/L), a mixture of yeast extract and peptone powder as the nitrogen source (5 g/L each). Both were added to distilled water and mixed until dissolution. Afterwards the solution was boiled at 100°C for 10 min. The scoby involving bacteria *Acetobacter* (acetic acid bacteria) was added into the HS medium for static cultivation at 26°C for 8 days. Based on previous studies, BC was formed for 7 days or more [28, 29]. When cultured for 8 days stably the BC gel thickness was about 1 cm. The BC gel samples were then recovered with 3% NaOH solution at 25°C using shaking water bath at 50 rpm for 90 min, neutralized with distilled water adjusted to pH 3.0 using acetic acid for 30 min. In addition, the BC gel samples were bleached with a 5% H_2_O_2_ solution at 90°C using a shaking water bath at 110 rpm for 60 min. After bleaching, the BC were dried in a drying convection oven (OF-21, Jeio tech Co.) at 35°C [27].

### Coating of BC by “*in situ*” laccase-assisted synthesis of polyaniline

The bleached BC gel samples were cut into small pieces (1.5 cm^2^) and placed in a 100 mL flask containing a mixture of 25 U/ml of laccase, 10 mM HBT mediator and 50 mM of aniline, in a final volume of 20 mL of acetate buffer (pH 4). The samples and solutions were stirred for 24 hours using two distinct temperatures, 4 ºC and room temperature, in a water bath (Grant, United Kingdom). The same procedure was also performed using an ultrasonic bath (USC600TH, VWR International Ltd., USA; frequency 45 kHz and power of 120 W) for 2 hours using the same reactional conditions. At the end of each experiment, the BC samples were washed thoroughly with distilled water to remove the by-products and the remaining starting reagents. Afterwards the BC samples coated with polyaniline were dried in a convection oven (OF-21, Jeio tech Co.) at 35°C for 24 hours.

### Polymers characterization

#### UV/Visible spectroscopy

The polymerization of aniline was followed by UV-Visible spectroscopy using a 96-quartz microplate reader (SynergyMx, Shimadzu, Japan) in the wavelength range of 230–700 nm.

#### ^1^H NMR and MALDI-TOF

The ^1^H NMR spectroscopy of polyaniline was determined using a Bruker Avance 400 (400 MHz). CDCl_3_ was used as deuterated solvent, using the peak solvent as internal reference. The polymer products obtained were also characterized by MALDI-TOF mass spectrometry using a Bruker Autoflex Speed instrument (Bruker Daltonics GmbH) equipped with a 337 nm nitrogen laser. The matrix solution for MALDI-TOF measurements was prepared by dissolving a saturated solution of 2,5-dihydroxybenzonic acid (DHB) in TA30 solution. Samples were spotted onto a ground steel target plate (Bruker part *n°* 209519) and analyzed in the linear negative mode by using factory-configured instrument parameters suitable for a 0.4–3 kDa m/z range (ion source 1: 19.5 kV; ion source 2: 18.3 kV). The time delay between laser pulse and ion extraction was set to 130ns, and the laser frequency was 25 Hz. The *M*_*n*_ (number-average molecular weight) and *M*_*w*_ (weight-average molecular weight) of polyaniline obtained after oxidation was obtained by MALDI-TOF direct analysis and according to the equations:

$$M_{n}=\frac{\sum n_{i}M_{i}}{\sum n_{i}}$$ Eq 1

$$M_{w}=\frac{\sum n_{i}M_{i}^{2}}{\sum n_{i}M_{i}^{2}}$$ Eq 2

Where *n*_*i*_ is the relative abundance of each peak; *M*_*i*_ is the *m/z* correspondent to each peak [16].

### BC surface coating characterization

#### FTIR-ATR

To analyze the chemical structure of BC polymerized with different conditions, FTIR-ATR spectra were obtained using an ATR FTIR, IRAffinity-1S (SHIMADZU). Scans were completed between 4000 and 400 cm^-1^ at a resolution of 8 cm^-1^, using 45 scans. Baselines for each sample spectrum were normalized using spectrum software.

#### Thermal analysis

Thermogravimetric analysis (TGA) measurements of the polymerized BC were carried out in a Perkin Elmer, TGA 4000 equipment, using about approximately 8–10 mg samples over the temperature range, 0–800°C at a heating rate of 10°C/min under a nitrogen flow of 20 ml/min. TGA was measured with the nanocomposite films placed in a high quality nitrogen (99.5% nitrogen, 0.5% oxygen content) atmosphere to prevent unwanted oxidation.

#### Swelling capacity

The monitoring of the membrane’s weight increase in distilled water was conducted at room temperature, during 48 h. The samples were removed from the water and their surfaces were carefully wiped with dry filter paper for weight purposes. The swelling ratio (SW) was then determined from Eq (3), where Ws and Wd are the weight of the swollen and dried membrane respectively [30].

$$Sw(\%)=\frac{\left(W_{s}-W_{d}\right)}{W_{d}}\times 100$$ Eq 3

#### Scanning electron microscopy

The coated BC samples were characterized using a desktop scanning electron microscope (SEM) coupled with energy-dispersive X-ray spectroscopy (EDS) analysis (Phenom ProX with EDS detector (Phenom-World BV, Netherlands)). All results were acquired using the ProSuite software integrated with Phenom Element Identification software, allowed for the quantification of the concentration of the elements present in the samples, expressed in either weight or atomic concentration. Prior to SEM analysis the samples were added to aluminium pin stubs with electrically conductive carbon adhesive tape (PELCO Tabs), with the excess removed using compressed air. Samples were coated with 2 nm of Au for improved conductivity. The aluminium pin stub was then placed inside a Phenom Standard Sample Holder, and different points for each sample were analysed for elemental composition.

#### Color evaluation of BC

The color (H), brightness (V), and saturation (C) of the polymerized BC were determined using Munsell's colorimetric transformation method, by measuring the X, Y, and Z values of the sample using a computational color-matching (CCM) system (JX-777, Japan). The color data for the dried samples were determined using a CCM system and illuminant D_65_ with a 10° standard observer. The color strength (K/S) was calculated from the reflectance values using the Kubelka–Munk equation:

KS=(1−R)22R Eq 4

where *R* is the reflectance, expressed as a proportional value; *K* is the absorption coefficient; and *S* is the light-scattering coefficient. The *K/S* values were presented as checksum *K/S*. All measurements were performed at least 10 times.

#### Conductivity of BC coated samples

Electrical conductivities were measured with a Fluke 123 Scopmeter (20 Mhz) using two -point probe technique placing them under a pre-defined distance between. The conductivity was calculated according to the following equation:

$$\sigma =\frac{1}{\rho }(S.{cm}^{-1})$$ Eq 5

obtained from the calculation R = ρ (L/A), where R: resistance in ohm.cm; ρ: resistivity (cm); L: distance between electrodes (6 mm); A: area of material section (6*0.5 mm).

#### Current measurement

The current was measured by a Keithley 6487 Picoammeter/voltage source. The sourcemeter recorded the current outputs form the coated BC while applying different voltages and the signals were transmitted to a computer.

## Results and discussion

The production and optimization of non-woven bacterial cellulose material has been previously reported by us [27]. Major steps covering the production of non-woven bacterial cellulose are cultivation, washing and bleaching, post-processing and drying. Post-processing might involve the incorporation of molecules like dyes and/or polymers [23]. Small or large molecules can be entrapped inside the wet BC fibers during post-processing and the subsequent drying conduct to their retention inside bacterial cellulose non-woven material for several applications [24, 31].

Herein, our goal was to entrap polyaniline into wet BC and coat the surface through the *in situ* polymerization of aniline by laccase. Aniline and laccase are expected to penetrate deeply inside the inner BC network porous structure and the oxidation can proceed using specific conditions namely low pH, in the presence of mediator (1-hydroxybenzotriazole- HBT). Moreover, coated BC composite membrane would be flexible and conductive, with potential for applications on wearable devices.

### Laccase-assisted polymerization of aniline

#### UV/Visible spectroscopy

**Fig 1:**
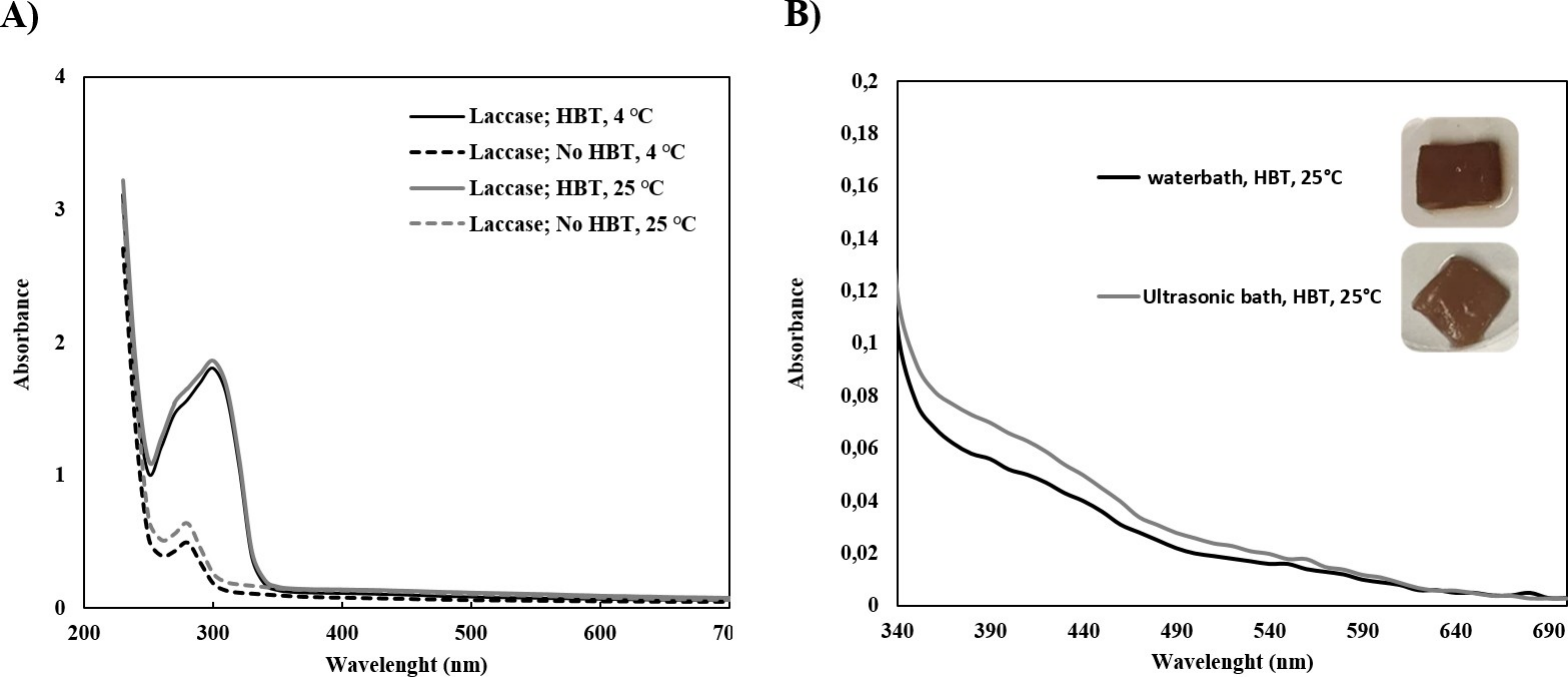
UV-Visible spectra of polyaniline solutions in presence of HBT after polymerization with laccase: **A)** spectra of polyaniline solutions at 4°C and 25°C, with and without mediator at wavelength between 230–650 nm, using a water bath as reactor; **B)** spectra of polyaniline between 340–690 nm after polymerization using both water bath **(a)** and ultrasonic bath **(b)** reactors.

Fig 1A shows the effect of mediator HBT on the UV-Visible absorption spectra of aniline solution after polymerization by laccase, using both water bath and ultrasonic bath reactors. We verified that the maximum absorption wavelength changed from 280 to 310 nm when using mediator, indicating aniline polymerization like previously reported [32]. It is also noteworthy the similar results obtained for both temperatures, 4°C and 25°C, used for polymerization which lead us the choose 25°C to proceed the further experiments.

From UV/Visible spectra (Fig 1B) one can evaluate the role of the reactors on the polymerization efficiency. One can observe a different spectral behavior depending on the reactor used. The spectra intensity of the solution is lower when using the water bath, however high BC coloration can be observed. The BC coloration is attributed to the insoluble material entrapped inside the BC fibrils and at their surface and low content of soluble oligomers/polymers can be detectable by UV/Visible spectroscopy. The opposite is observed for ultrasonic bath reactor. In this case, slightly higher amount of soluble oligomers/polymers is detected in solution however similar coloration of the BC samples was observed. This give as indication of the positive role of ultrasound as potentiating the polymerization leading to the production of higher amount of material, either on soluble and/or insoluble form.

### Characterization of the polymer

#### ^1^H NMR

**Fig 2:**
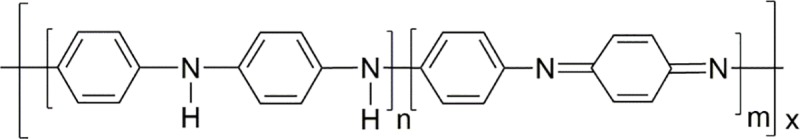
Representation of the proposed structure of polyaniline after laccase-assisted polymerization mediated by HBT [25].

The ^1^H NMR of aniline is represented by three peaks, a duplet at δ_H_ 6.71 ppm, followed by two triplets, one at δ_H_ 6.77 and the other at δ_H_ 7.17 ppm. Comparing the ^1^H NMR of aniline monomer with its spectrum after polymerization, it is possible to perceive the presence of new peaks at δ_H_ 7.46 ppm (S1 Fig). These new peaks are duplets, with coupling constants of *J* = 6.8/6.4 Hz, which are typical of *ortho* coupling, suggesting that polymerization occurred via radical attack of the nitrogen to the *para*-position, giving rise to a polymer with the structure suggested in Fig 2.

#### MALDI-TOF

**Fig 3:**
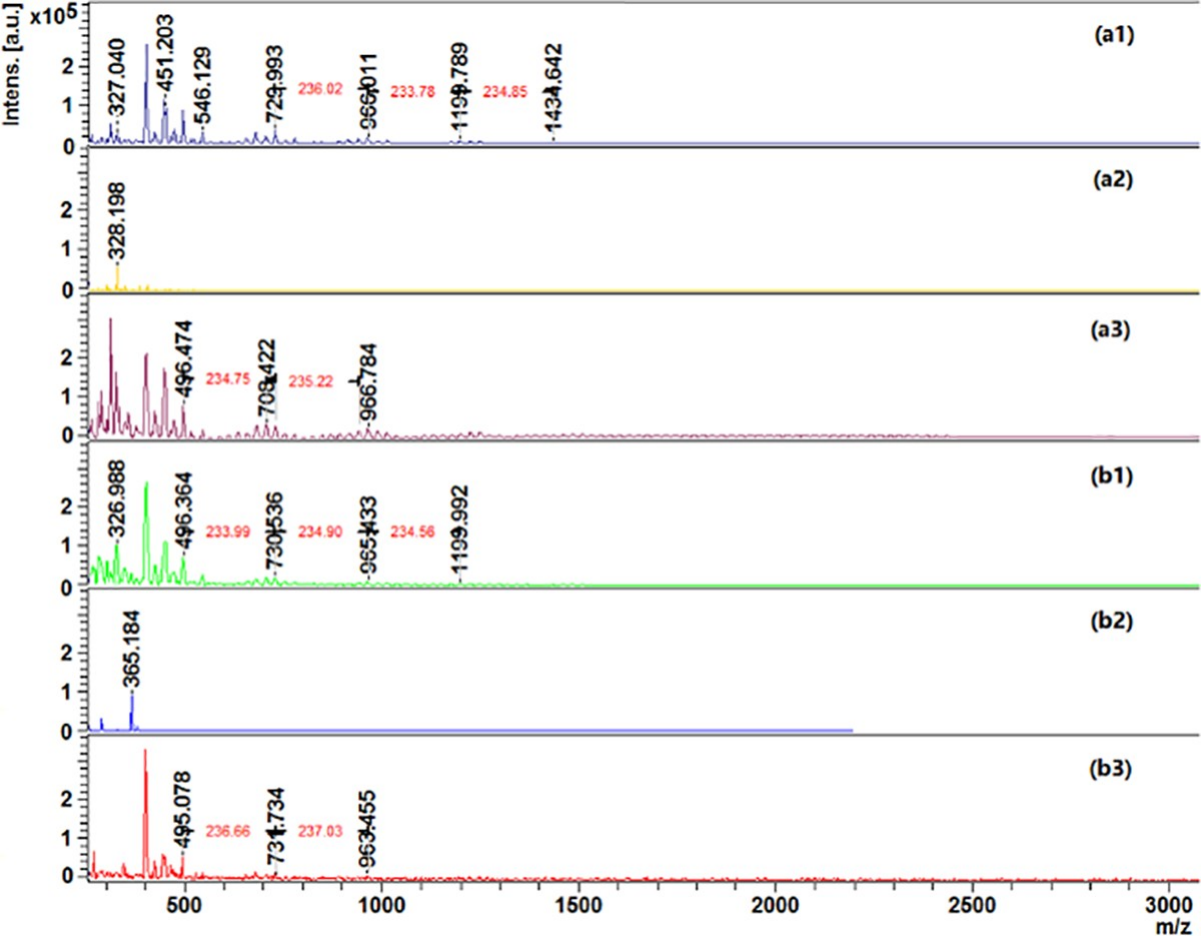
MALDI-TOF analysis of polyaniline polymerized by laccase: **a1)** aniline + laccase + HBT (WB); **a2)** aniline + without laccase + without HBT (WB); **a3)** aniline + without laccase + HBT (WB); **b1)** aniline + laccase + HBT (US); **b2)** aniline + without laccase + without HBT (US); **b3)** aniline + without laccase + HBT (US).

The aniline polymerization was also confirmed by MALDI-TOF analysis (Fig 3). The collected data reveal a pattern of 234–236 *m/z*, which might be attributed to ionized aniline trimers, when laccase and HBT are used simultaneously. Only one peak corresponding to a trimmer has been detected when the reaction was conducted in the absence of laccase or in the absence of HBT mediator. One can also conclude that the reactors used for polymerization did not influence the size of the final products, which present similar ionization by MALDI-TOF.

From MALDI-TOF data one was able to characterize the new oligomers by calculating the *Mn*, *Mw* values and the degree of polymerization (DP) (Table 1). As mentioned before, no significant differences are obtained in terms of average DP when using the water bath or the ultrasonic bath, for aniline polymerization. On the other hand, and once again, the role of laccase and mostly the mediator are confirmed by these results. Without mediator it is possible to polymerize aniline however shorter oligomers are obtained. It is noteworthy that in the absence of laccase and using HBT, the polymerization of aniline occurred. The oxidative process might take place without the need of the catalyst, helped by the radical formation under an ultrasonic field.

**Table 1:** Polyaniline characterization in terms of *Mn*, *Mw* and average polymerization degree (DP) using water bath and ultrasonic bath for reaction, evaluated by MALDI-TOF.

	Water bath (WB)			Ultrasonic bath (US)		
	*Mn*	*Mw*	DP	*Mn*	*Mw*	DP
**Aniline + laccase + No HBT**	565	585	6	525	541	5
**Aniline + No laccase + HBT**	282	293	3	650	702	7
**Aniline + laccase + HBT**	930	1000	10	893	925	9

### Characterization of the coating

#### FTIR

**Fig 4:**
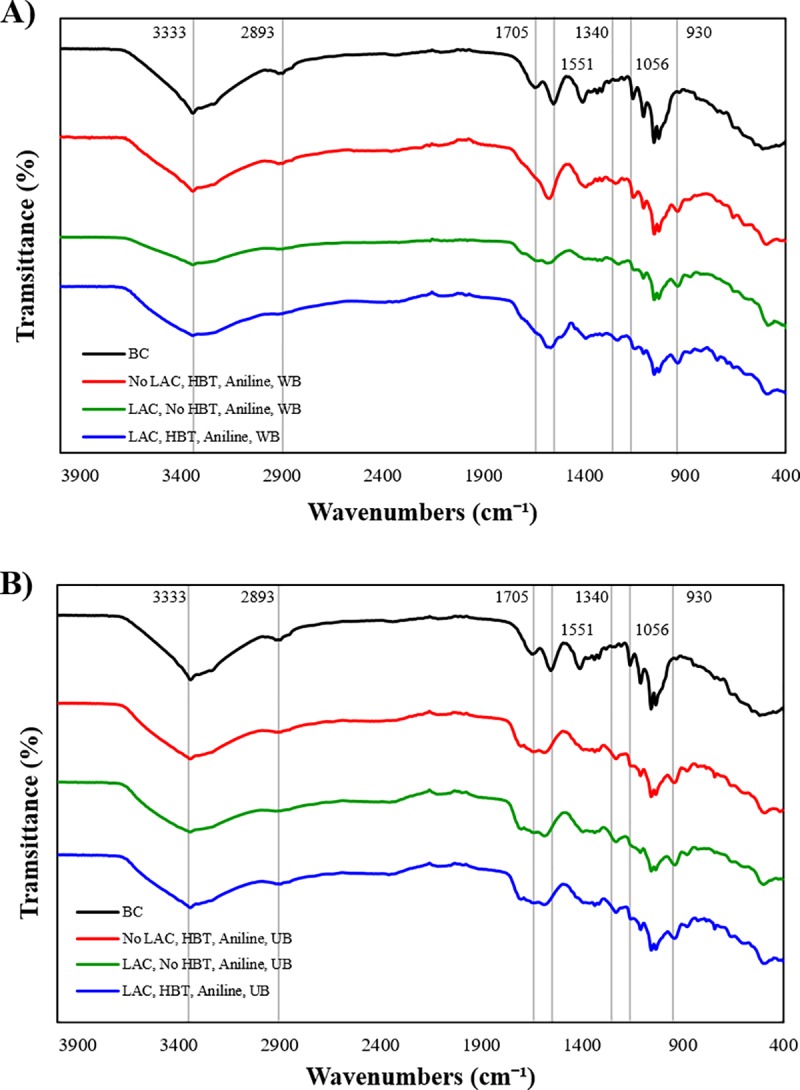
FTIR-ATR analysis of polyaniline polymerized by laccase; **A)** the polymerization was conducted in a water-bath (WB); **B)** the polymerization was conducted in an ultrasonic bath (US).

The FTIR-ATR of BC coated with polyaniline through different reactional procedures is observed in Fig 4. The spectra of bleached BC shows absorption peaks at ν 3333, 2893, 1551 and 1056 cm^-1^. These peaks are attributed to the stretching vibrations of different parts of the BC: ν 3333 cm^-1^ (-OH), ν 2893 cm^-1^ (-CH_2_) and ν 1056 cm^-1^ (-C-O), and the bending vibration of the C-H at ν 1551 cm^-1^. The BC coated with polyaniline show a new peak at ν 930 cm^-1^ which correspond to the bending vibration of -C = C bonds, and is attributed to the aromatic units of polyaniline. Around ν 1340 cm^-1^ a broader band is observed, which corresponds to the stretching vibration of aromatic amines (-N-C). Between the region at ν 1570 and 1705 cm^-1^ another broad band is observed. In this area are observed the stretching vibration of quinoid and benzoid structures. When aniline is polymerized *in situ* with BC, the coating results from two major events, namely the hydrogen bonding between the hydroxyl groups of BC and the polyaniline chain and the electrostatic interactions between the negatively charged BC and the positively charged polyaniline. The protonation of the polyaniline imine nitrogen through interactions with the hydroxyl groups of the BC nanofibriles strengthened the interactions between the polyaniline and BC [25]. The spectra obtained are in accordance with the spectra presented in literature [33].

#### Morphology

**Fig 5:**
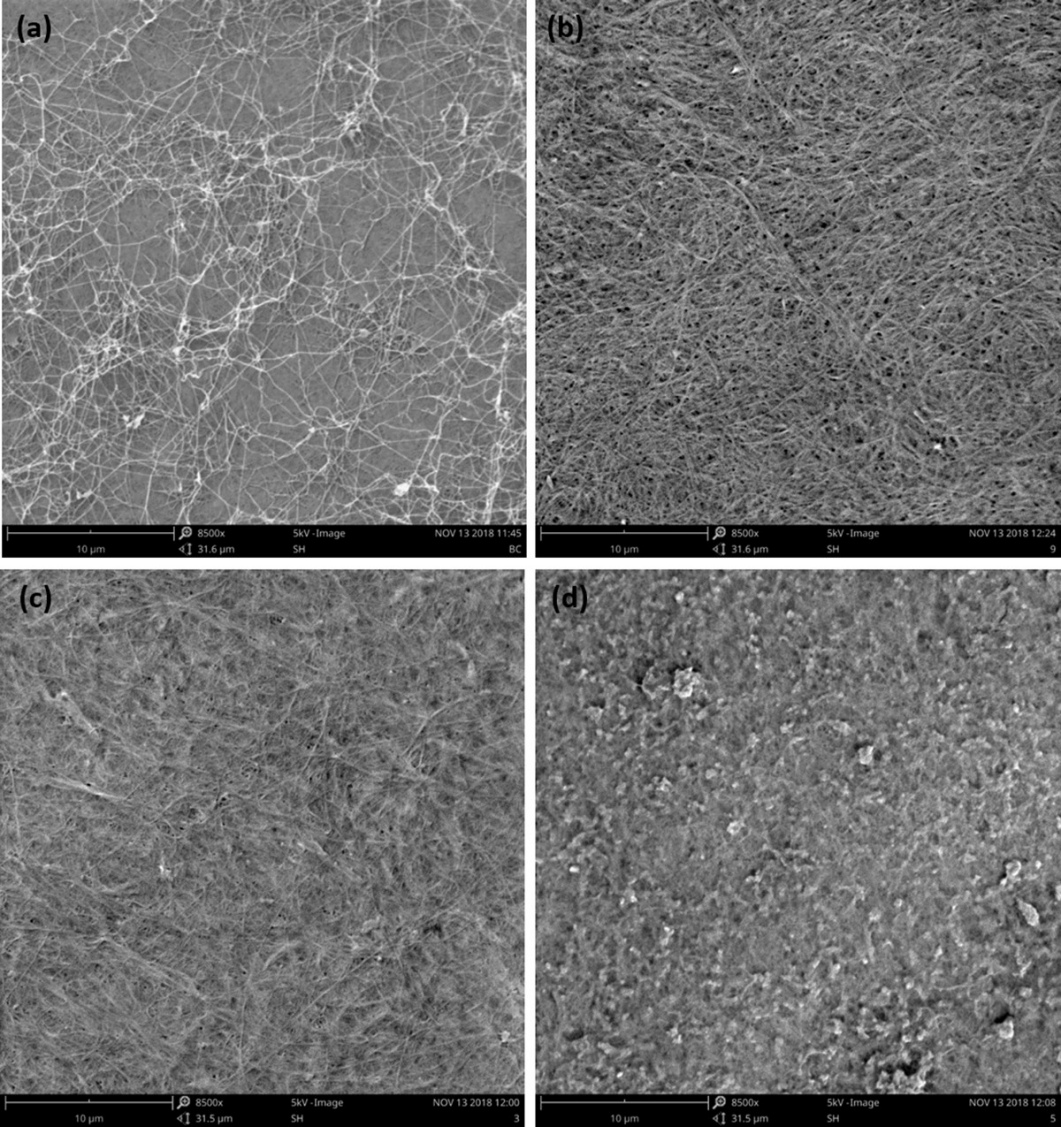
SEM photographs (x 8500) of BC samples coated with polyaniline: (a) bleached BC; (b) aniline + HBT + without laccase; (c) aniline + laccase + without HBT; (d) aniline + laccase + HBT; the samples were all incubated in a water-bath (WB).

SEM analysis was carried out in the current study aiming to observe the BC surface after coating with polyaniline. As it can be seen in Fig 5, bleached BC presents entangled fibrils which are less evident as the coating is undertaken. The presence of polyaniline can be detected especially when the combined laccase/mediator system is applied (Fig 5D). The surface is less porous and a more smooth and compact structure take place when the conditions of aniline polymerization are favored *in situ*. In this case the coated samples present flakes/granules, and nearly absence of fibers at the surface.

#### Thermal degradation stability

**Fig 6:**
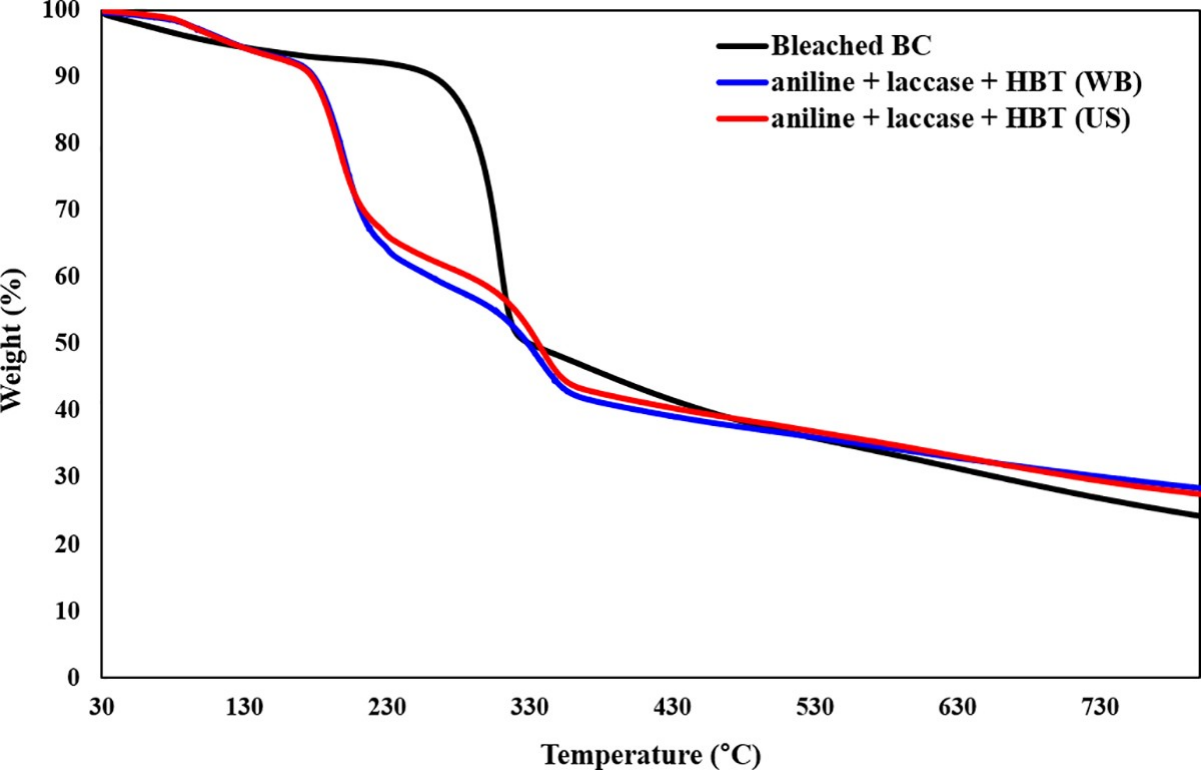
TGA analysis of polyaniline polymerized by laccase: (a) untreated BC; (b) bleached BC; (c) aniline + laccase + HBT (WB); (d) aniline + laccase + HBT (US).

The TGA curves of coated BC samples were measured at heating rate of 10°C/min, and is shown in Fig 6. The TGA curves of bleached BC and polyaniline coated BC are shown for comparison. The TGA curve of bleached BC presents the typical thermal behavior loosing close to 75% of the initial weight at 750°C. The TGA curves of coated BC with polyaniline, either using a water bath or an ultrasonic bath as reactors, show weight loss in three main stages. The weight loss observed within 100°C was assigned to the removal of moisture present in the nanocomposite samples. The first stage of weight reduction at approximately 200°C might be due to the combination of bacterial cellulose and the side chains or impurities of polyaniline. At this stage, the bleached BC and the coated BC revealed different thermal behavior. The higher weight loss observed for the coated BC might be related to disruption of the intermolecular hydrogen bonds of cellulose suggesting the rearrangement of the macromolecular structure. This sharp weight loss might be also ascribed to the eradication of the crystalline part of BC and decomposition of amorphous BC into a monomer of ᴅ-glucopyranose and further into a free radical. The second stage of weight loss occurred at approximately 300°C due to thermal-oxidative degradation of the main polyaniline chain. All the tested samples loosed close to 75% of the initial weight above 750°C. Since thermal degradation is affected by structural parameters like molecular weight, crystallinity and orientation, the results obtained are in accordance with these assumptions revealing a shift of temperature degradation for samples coated with polyaniline [5, 34, 35].

#### Swelling capacity analysis

**Fig 7:**
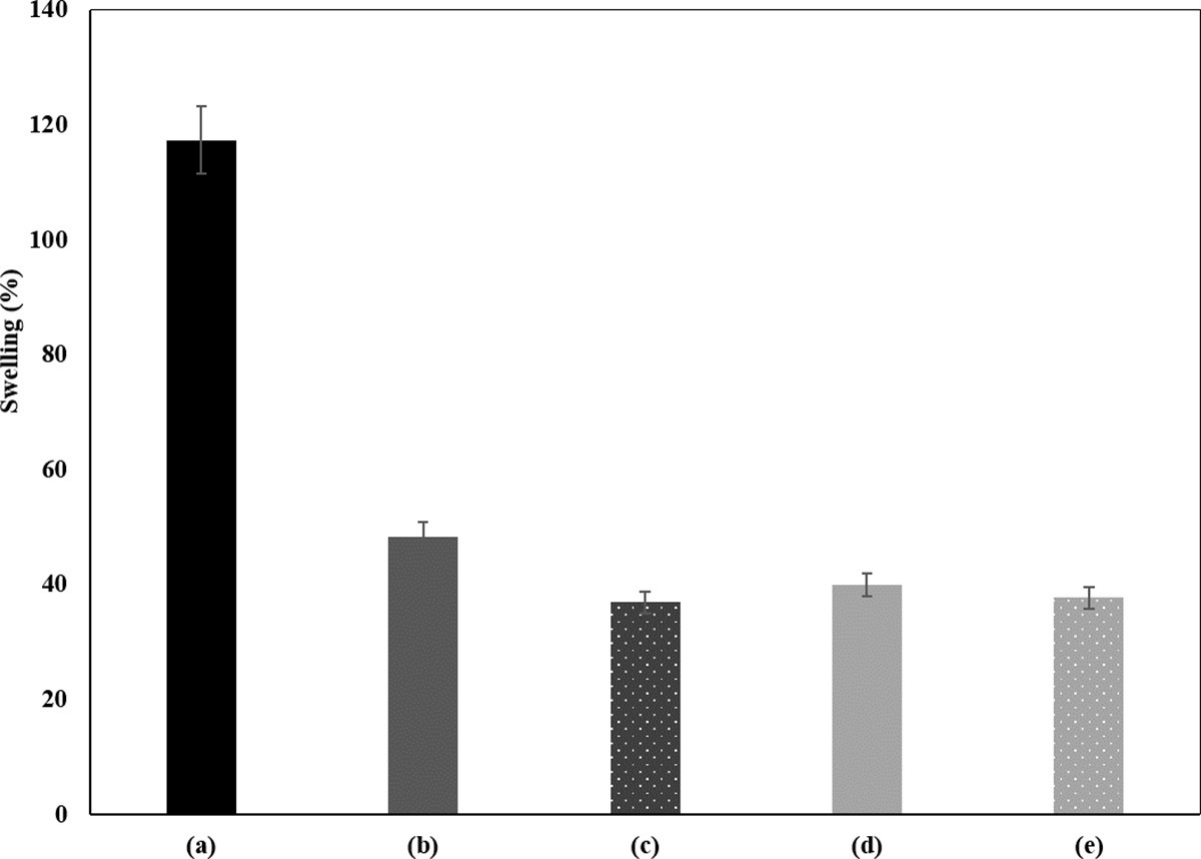
Swelling capacity analysis of polyaniline polymerized by laccase: (a) bleached BC; (b) aniline + without laccase + HBT (WB); (c) aniline + laccase + HBT (WB); (d) aniline + without laccase + HBT (US); (e) aniline + laccase + HBT (US).

The hydrophilic nature and water retention capacity of BC is influenced by the fibril arrangement and high surface area per unit mass [36]. However, molecules entrapment and processing conditions might alter this arrangement and disturb BC behavior. Herein, the swelling studies were conducted aiming to study the effect of BC matrix and polymerization method on the water retention capacity of the differently treated samples. The evaluation of this property is demonstrative of the extent of the PANi entrapment inside BC pores which influence the samples behavior when in contact with water. The hydrophobicity acquired by the samples is favorable depending on the final applications envisaged. Fig 7 presents the swelling capacity of polyaniline polymerized by laccase. The untreated and bleached BC samples present high swelling capacity over 110%. With polyaniline incorporation, a significant decrease in the swelling capacity was observed under 60%, which might be attributed to the obstruction of the BC pores by polyaniline as well as to its hydrophobic nature [30]. The swelling capacity of samples coated in the presence of laccase is slightly lower confirming the higher amount of polyaniline entrapped inside BC that hinder the water absorption.

#### Conductivity of coated BC

**Fig 8:**
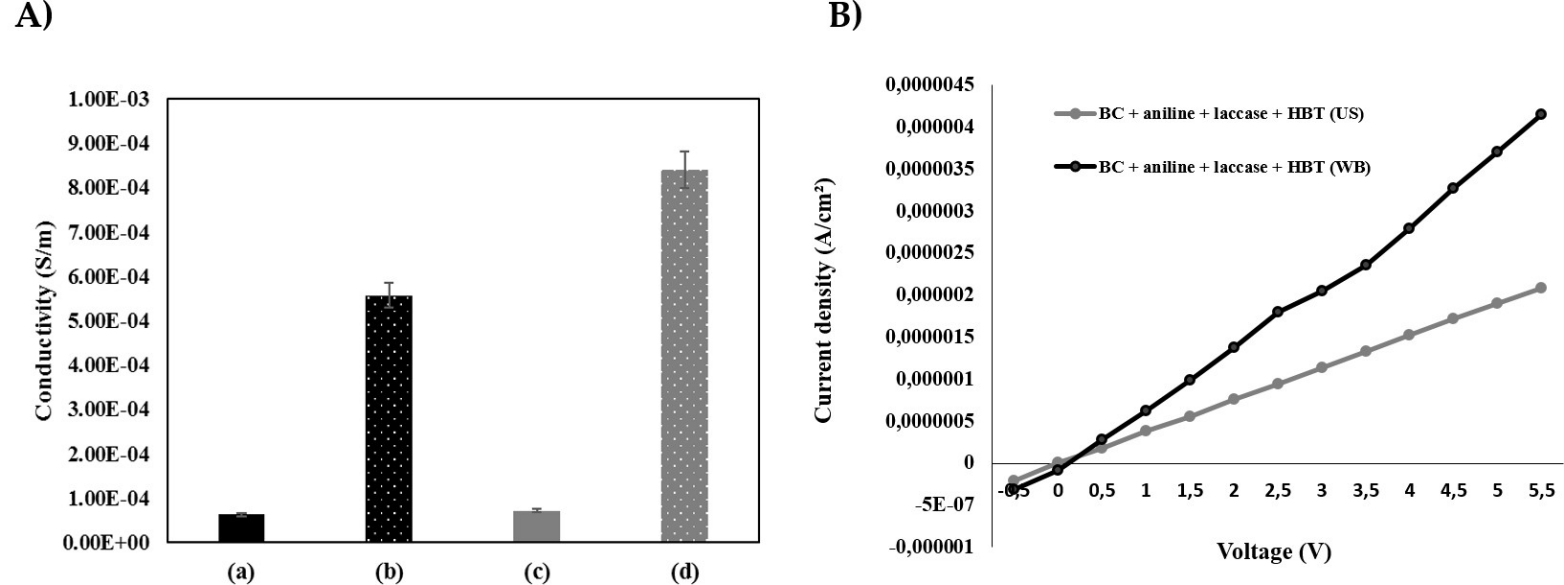
**A)** Conductivity of the aniline after polymerization under different conditions; **(a)** Aniline + without laccase + HBT in water bath; **(b)** Aniline + with laccase + HBT in water bath; **(c)** Aniline + without laccase + HBT in ultrasonic bath; **(d)** Aniline + with laccase + without laccase in ultrasonic bath; **B)** Current density of aniline after laccase-assisted polymerization onto BC using different reactors: (a) Aniline + laccase + HBT, water bath; (b) Aniline + laccase + HBT (US).

The conductivity of coated BC samples was evaluated and the results are presented in Fig 8. From the data obtained one can highlight that samples incubated in the absence of laccase or HBT mediator, either using the water bath or the ultrasonic bath, did not show conductive character. The samples coated by polyaniline assisted by laccase in the presence of HBT mediator reveal a conductive behavior. These results are in accordance with the UV/Visible data which reveal higher polymerization when the enzymatic reaction is assisted by a mediator. The data obtained allowed us to perceive that the use of ultrasounds influenced the conductive character of the coated samples. As previously stated by others, the enzymatic processes are potentiated by ultrasound due to mass transfer phenomena involved, increasing the delivery of the substrate to the active site of the enzymes, and subsequently increasing their catalytic efficiency [37]. In aniline polymerization with laccase and mediator, ultrasonication seems to play a positive effect since high levels of conductivity are perceived on the samples treated under these conditions.

The current density variation with current voltage was evaluated for the samples coated with polyaniline polymerized *in situ* using both water bath and ultrasonic bath reactors (Fig 8B)). The current density of BC samples coated with polyaniline reveal a linear tendency. The higher the slope of the linear curve higher is the sample’s conductivity, which is tendentially higher for samples incubated under ultrasound medium. The effect of mass transfer inherent to ultrasound devices may lead to the transport of the materials inside the BC structure to positions that favor the electric conductivity. Moreover, and despite the low DPs obtained when using these devices, the amount of insoluble materials into the inner spaces of the BC network would confer more conductivity to the coated materials.

#### *In situ* coloration of BC with polyaniline

**Fig 9:**
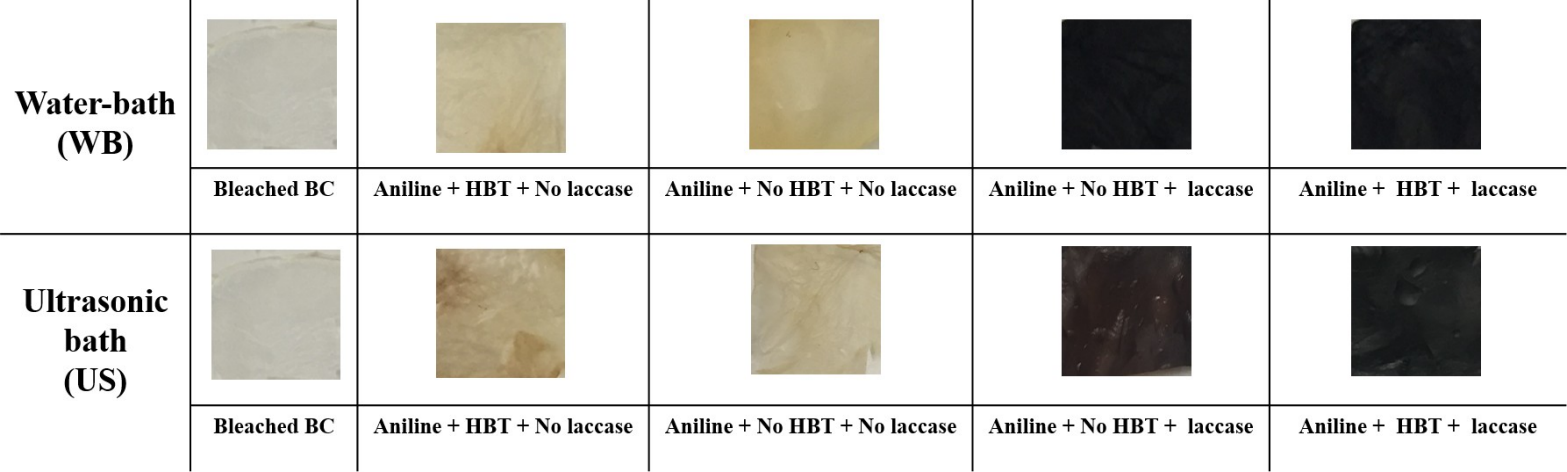
Photographs of BC samples after *in situ* aniline polymerization under different conditions.

Besides the conductive character conferred by the polymerized aniline, the BC samples acquire also coloration after the *in situ* polymerization of the monomer (Fig 9). In S1 Table is presented the spectral characterization of BC samples after aniline polymerization. The color strength, visually evaluated, corroborates the MALDI-TOF and the conductivity results obtained. Higher degrees of polymerization correspond to higher color strength (*K/S*) of the BC coated samples. As previously mentioned, the coloration of BC samples was higher when the reaction was conducted in a ultrasonic bath, corroborating the mass transfer phenomena involving within this device, which results in higher amount of polymer entrapped. The presence of HBT, as already stated, is crucial for the polymerization and therefore for coloration, which is intrinsically dependent on the amount and size of the polymers produced. The differences between samples incubated in the absence and in the presence of mediator are also evidenced by chroma analysis (S1 Table).

## Conclusions

In this study we investigated the ability of laccase to *in situ* polymerize aniline in the presence of HBT giving rise to a colored and conductive material. The optimum conditions for *in situ* polymerization were established for the highest amount of polymer produced with the highest polymerization degree (2 h at pH = 4; 25°C; US; HBT). The reactors used to conduct the polymerization, as well as the HBT mediator, influenced greatly the amount and size of the polyaniline as well as the level of coloration of the BC samples. Ultrasonic bath reveal to be the most prone device to use as polymerization reactor since it allowed to obtain the highest coating levels which proportionally lead to higher conductivity. The *in situ* enzymatic polymerization of aniline onto BC supports reveal to be a green methodology to confer at the same time conductivity character and coloration, opening up new routes for the functionalization of these promising cellulosic materials.

## Supporting information

S1 Fig ^1^H NMR of polyaniline after polymerization with laccase (US, 2 hours, 25°C). (DOCX)

S1 Table Spectra analysis of BC samples coated with polyaniline. (DOCX)
