# Supplementary figures and images for: Conductive bacterial cellulose by in situ laccase polymerization of aniline
Source: PLoS One. 2019 Apr 15;14(4):e0214546. doi: 10.1371/journal.pone.0214546 (PMC6464183; doi:10.1371/journal.pone.0214546)

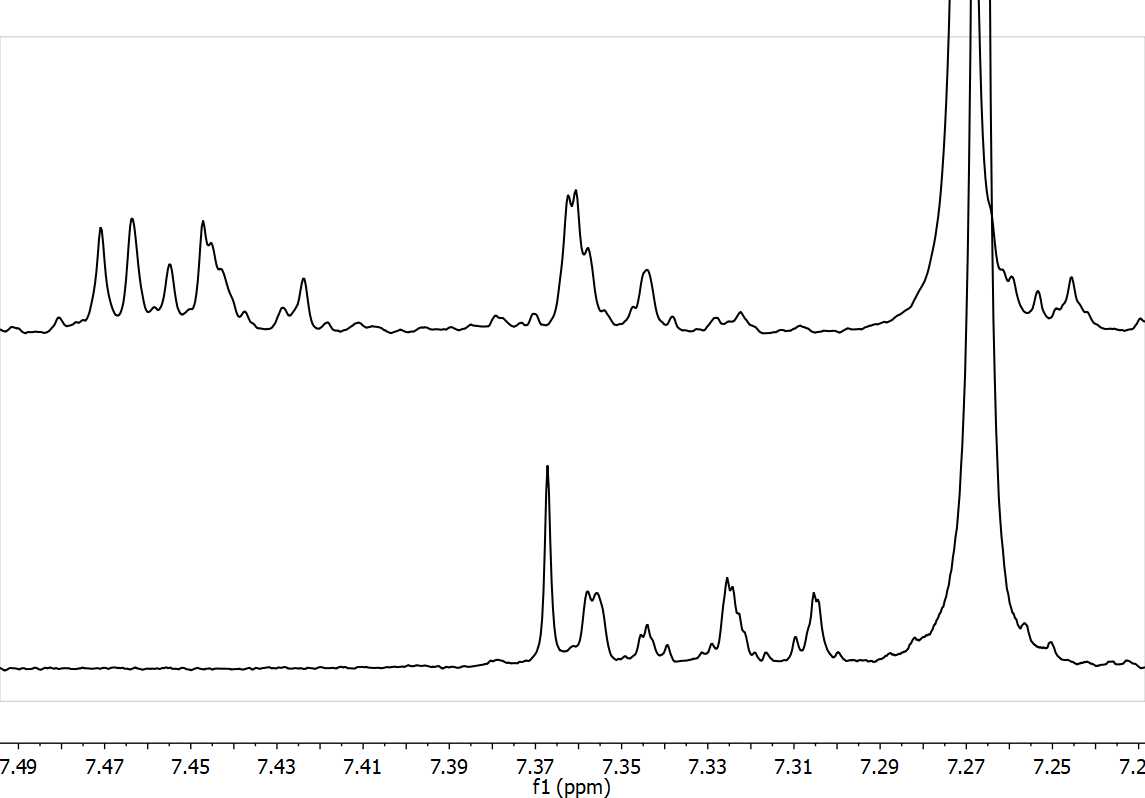


**F**

**S1 Fig.** 1H NMR of polyaniline after polymerization with laccase (US, 2 hours, 25ºC).

Supplement: S1 Fig — (DOCX) [file pone.0214546.s001.docx]
