# Supplementary material for: Conductive bacterial cellulose by in situ laccase polymerization of aniline
Source: PLoS One. 2019 Apr 15;14(4):e0214546. doi: 10.1371/journal.pone.0214546 (PMC6464183; doi:10.1371/journal.pone.0214546)
Supplement: S1 Table — (DOCX) [file pone.0214546.s002.docx]

**S1Table.** Spectra analysis of BC samples coated with polyaniline

| **Samples** | **Checksum *K/S*** | | **Hue** | **Value** | | **Chroma** |
| --- | --- | --- | --- | --- | --- | --- |
| **(a) Aniline + laccase + HBT, in water bath** | | 157.98 | 3.7YR | | 3.79 | 2.87 |
| **(b) Aniline + without laccase + HBT, in water bath** | | 40.03 | 1.1Y | | 6.14 | 3.24 |
| **(c) Aniline + laccase + HBT, in ultrasonic bath** | | 315.44 | 4.6YR | | 2.65 | 0.36 |
| **(d) Aniline + without laccase + HBT, in ultrasonic bath** | | 64.85 | 3.5Y | | 5.22 | 1.42 |

*Hue: The dimension which distinguishes color group
*Value: The lightness of a color
*Chroma: The degree of departure of a color from a grey having the same Munsell value
